# An extended SEIRDV compartmental model: case studies of the spread of COVID-19 and vaccination in Tunisia and South Africa

**DOI:** 10.1097/MS9.0000000000000627

**Published:** 2023-04-18

**Authors:** Phemelo Tamasiga, Helen Onyeaka, Great C. Umenweke, Olivier Uwishema

**Affiliations:** a Am Depenbrock, Gütersloh, Germany; b School of Chemical Engineering, University of Birmingham, Edgbaston, Birmingham, UK; c Federal University of Technology Owerri, Nigeria; d Department of Project and Education, Clinton Global Initiative University, New York, NY; e Department of General Medicine, Faculty of Medicine, Karadeniz Technical University, Trabzon, Turkey; f Department of Research and Education, Oli Health Magazine Organization, Kigali, Rwanda

**Keywords:** COVID-19, SEIR model, SEIRDV model, SIR model, Tunisia, vaccination, South Africa

## Abstract

In the wake of the unprecedented health crisis triggered by the global COVID-19 pandemic, countries are still grappling with the pandemic’s immediate health and socioeconomic consequences. This paper presents an extended SEIRD model with vaccination to study the evolution of COVID-19 in South Africa and Tunisia since the commencement of the vaccination campaign in each country, respectively. Epidemiologists often quantify a risk reduction following the implementation of non-pharmaceutical containment measures and vaccines when attempting to stem the spread of pandemics. However, an important question they often ask is the effectiveness of the non-pharmaceutical containment measures (social distancing and lockdowns) and the efficacy of such measures, including vaccines. Africa’s COVID-19 vaccine roll-out stands at 16% as of April 2022; however, the continent lags behind many developing countries even though it harbours about 16% of the world population. While proliferating literature quantifies the efficacy and effectiveness of COVID-19 vaccines, very little has been done using the SEIRDV model in African countries. This study compares the model-predicted results with the available data to estimate the dynamics of the infected population, using data from 20-03-2021 to 30-12-2021. A simulation of the SEIRDV model is performed and fitted to the data. Simulating the model involves solving a system of Ordinary Differential Equations numerically by taking the initial values for the key model parameters as inputs. After simulating the SEIRDV model, the model parameters are compared with real-world COVID-19 and vaccination data in order to estimate the values of the different parameters that best fit the observed data. The results of the study showed an inverted U-shaped trend for the infection rate after vaccination, indicating that increasing the vaccination rates reduces the transmission rates. Therefore African countries must continue to scale up the vaccination campaigns, and the world needs to endeavour to ensure an equitable vaccination roll-out to developing countries.

## Introduction

Highlights

- This paper presents an extended SEIRD model with vaccination for studying the evolution of the COVID-19 in South Africa and Tunisia since the commencement of the vaccination campaign in each country, respectively.
- Epidemiologists often quantify a risk reduction following the implementation of containment measures and vaccines when attempting to stem the spread of pandemics.
- Africa’s COVID-19 vaccine roll-out stands at 16% as of April 2022; however, the continent lags behind many developing countries even though it harbours about 16% of the entire world population.

In December 2019, the first case of the novel severe acute respiratory syndrome coronavirus 2 (SARS-CoV-2) was detected in Wuhan, China^1,2^. The transmission of SARS-CoV-2 has been speculated to be associated with three primary mods namely; ’contact’, ‘droplet’, and ‘airborne’ transmission^3^. Statistics have shown that more than 5.63 million deaths and 362 million cases have been recorded. The pandemic burdened the world’s socioeconomic and health systems^4–6^. But, more importantly, the pandemic exposed the inherent structural deficiencies of health systems, especially in developing countries.

**Figure 1:**
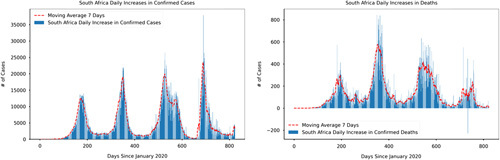
Daily increases confirmed and death cases in South Africa. Source: Author’s calculations and visualisations based on data collected from the official website of Johns Hopkins University (2020): https://github.com/CSSEGISandData/COVID-19 (Accessed April 24 2022).

**Figure 2:**
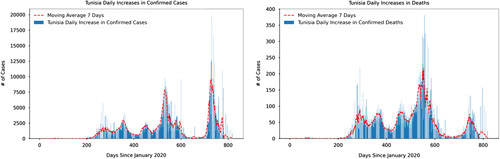
Daily increases in confirmed and death cases in Tunisia. Source: Author’s calculations and visualisations based on data collected from the official website of Johns Hopkins University (2020): https://github.com/CSSEGISandData/COVID-19 (Accessed April 24^t^ 2022).

**Figure 3:**
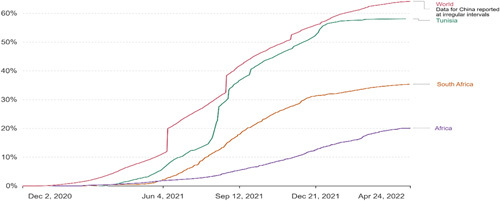
Share of people who received atleast one dose of COVID-19 vaccination protocol (total number of people who received atleast one vaccine dose divided by the total population of the country). Source: Our World in Data, Coronavirus (COVID-19) Vaccinations—Our World in Data (Accessed April 24th 2022).

**Figure 4:**
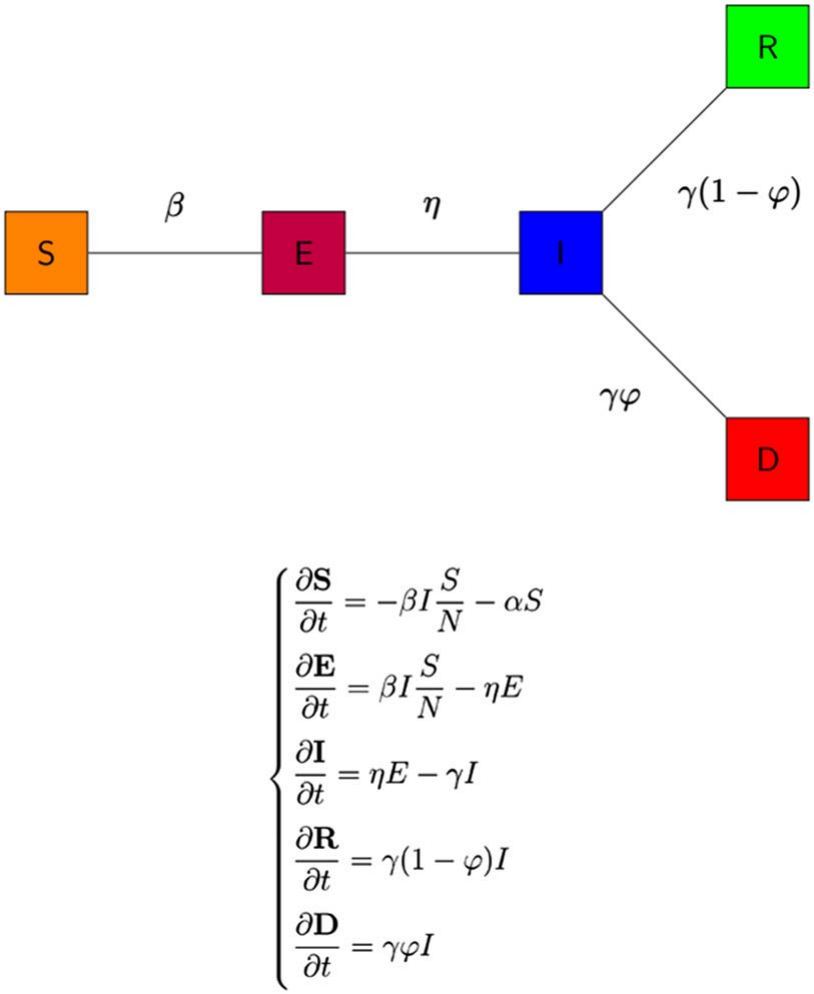
Illustration of the SEIRD Model. Source: Authors illustration based on Piccolomini and Zama^26^.

In response to the spread of the COVID-19 pandemic, governments around the world implemented non-pharmaceutical containment measures such as social distancing, lockdowns, shutting down public gathering activities, closing down both private and public schools, testing and tracing protocols and extending periods of state of emergencies^7^. Whilst the adoption of such policy measures to reduce the number of infections has been implemented in many countries, statistics have shown varying degrees of the number of cases amidst the implementation of such non-pharmaceutical containment measures. One of the plausible explanations for the differences is that COVID-19 containment measures have largely been met with compliance and adherence challenges such as weak government authority, misinformation and high population densities^8–10^. On the bright side, these non-pharmaceutical containment measures have started to show a reduction in COVID-19 confirmed and death cases. Figures 1–4 show the daily time series data of COVID-19 (confirmed and death cases) for Tunisia and South Africa, respectively^1^ 1The data was collected from the official website of Johns Hopkins university (2020): https://github.com/ CSSEGISandData/COVID-19

. Additionally, Figures 1–4 show a 7-day moving average indicated by the red dotted trend curve. The seven-day moving average indicates the daily number of newly diagnosed COVID-19 infections over a seven-day period. We quickly point out that due to delays in reporting, the reported confirmed and death cases displayed in the figures do not necessarily show the number of new confirmed and death cases on that day. For example, the actual death and confirmed cases may be under-reported due to limited testing capacities and challenges in establishing the cause of death.

Vaccines are a critical tool that is often used to manage pandemics and reduce mortality rates. Therefore, it is very important that there is equity, access, and safe distribution of effective vaccines in African countries. Vaccines in Africa were obtained by purchasing new doses and receiving them from donor countries such as China and India. The United Nations also provided African countries with vaccines such as AstraZeneca and BioNTech through its COVID-19 Vaccines Global Access (COVAX) initiative. High-income countries made bulk pre-orders of the COVID-19 vaccines, and COVAX is especially important in covering the COVID-19 vaccine access gap^12^. However, there is still a worrying divergence in the global COVID-19 vaccine roll-out between Africa and the rest of the world. As of the 8^th^ of March 2023, of the 13.3 billion vaccine doses administered worldwide, only 774.3 million vaccines have been administered in Africa, which translates into 17.18% of vaccines^13^. This is an eminent problem since Africa accounts for over 16% of the global population. Figure 3 below shows the evolution of vaccine administration in the case study countries South Africa and Tunisia. Additionally, the Figure 3 depicts the vaccination rate of the world and Africa.

Vaccine effectiveness, vaccine efficacy and vaccine coverage against COVID-19 are some of the main determinants of COVID-19 infection rates^14–16^. Factors that may affect vaccine efficacy include behavioural patterns, for example, older people could be less likely to get exposed to SARS-CoV-2 due to avoidance of social gatherings^17^. Vaccines and other factors related to spreading the disease should be considered, such as adherence to preventive measures. It was shown that the reduction in infection rate began with the administration of vaccines in different countries. As indicated in Figure 3 above, Africa has vaccinated about 20.10% of its population.

In contrast, the entire world has vaccinated about 64.21% of its population, a large proportion of these vaccinations occurred in developed countries^18^. The low vaccination rates in Africa threaten lives, affecting both Africa and global recovery^19^. In the set of countries considered in this study (at the time this simulation was conducted, 24 April 2022), the share of people who had received atleast one dose of COVID-19 vaccination in Tunisia was about 58.10%, and it was about 35.41% in South Africa^18^.

Mathematical models are often used to analyse the evolution of infectious diseases^20^. The outbreak of COVID-19 has triggered a renewed interest in employing these mathematical models. These models are useful in simulating the evolution of pandemics under different scenarios, such as social distancing and lockdowns. Moreover, these models vary in the assumptions made and different states of the population are considered. Consequently these models differ in their approaches for estimating parameters such as death rates, incubation periods and infection rates. The SIR model (susceptible, infected, and recovered) is one of the most implemented and pioneering epidemiological mathematical model developed by Kermack and McKendrick^21^. This model comprised three elements: susceptible, infected, and recovered. Many extensions of this basic model were developed to increase its complexity and include more compartments. For example, the SEIR model (susceptible, exposed, infectious, recovered). The SEIRD model (susceptible, exposed, infected, recovered and dead) is an extension that includes the death compartment^22,23^. The SEIRD model has been employed in hybrid epidemiology modelling studies such as Ala’raj *et al*.^24^. These models have been applied to infectious diseases and to study the diffusion of fake news. For example, D’Ambrosio *et al*.^25,26^ employed a SEIR model which is usually used in epidemiology to study the diffusion of fake information.

The aim of this paper is to compare the model-predicted results with the available data to estimate the dynamics of the infected population, using data from 20-03-2021 to 30-12-2021. Among the vaccine-related papers within the COVID-19 literature, we employ an extended SEIRD model with the compartment of vaccinated people hence the SEIRDV model (susceptible, exposed, infected, recovered, dead and vaccinated). This paper then models the interaction between different states in the same spirit as Wu and colleagues (2020), who asserted that the SEIR model simulation could explain the interaction between the model compartments. This study adopts a mathematical model fitted to data as in the study of Moore *et al*.^27^, who investigated non-pharmaceutical policies and vaccines. The study of Moore *et al*.^27^ is important because it uses a mathematical modelling approach to fit UK COVID-19 data and forecasts infections considering the vaccination rates over time. The authors concluded that although vaccination substantially reduced total deaths, it only provides partial protection and needs to be implemented together with non-pharmaceutical interventions such as social distancing and lockdowns. The inclusion of vaccines in a SEIRD model adopted in this paper is based on the paper by Piccolomini and Zama^11^, which investigated a SEIRD model with the infection rate taken as an inverse of the function of time to take into account the social distancing restrictions implemented by the Italian government. We extend the model by including the vaccines instead of the social restrictions.

The contribution of this study is three-fold. Firstly, during the outbreak of COVID-19, African countries did not have sufficient testing capacities, leading to insufficient data points. This study uses data taken from 20-03-2021 to 30-12-2021, which will reflect more reasonable and reliable results as opposed to early models where there was limited data due to limited COVID-19 testing being done. Secondly, unlike this study, existing epidemiological studies have not considered the vaccination dynamics for African countries^27^. Thirdly, to determine the reliability of the SEIRDV model, the simulated model parameters are compared to real-world COVID-19 and vaccination data.

The rest of the paper is organised as follows. The proposed COVID-19 model is formulated in Section 2, and data, and its sources are shown in Section 3. The results and analysis are presented in Section 4. A discussion to support theoretical results is presented in Section 5. Finally, the conclusion is provided in Section 6.

## Methodology

Similar to Brauer^28^, this study employs a deterministic compartmental model and solves a system of Ordinary Differential Equations (ODEs) using initial values of the ODE. To demonstrate the approach, the study utilises a benchmark SEIRD model (Susceptible, Exposed, Infections, Recovered, Dead) without vaccines and subsequently explores the SEIRDV model (Susceptible, Exposed, Infections, Recovered, Dead, Vaccinations). The primary difference between these models is the inclusion of a compartment for vaccinated individuals. As the current model is derived from Piccolomini and Zama^11^, the proof of the stability of the SEIRDV model is deferred to their publication. The SEIRDV model is then applied to the COVID-19 and vaccination data from South Africa and Tunisia, which were selected to provide variation in population size, economic size, and income classification based on the World Bank^10^.

### SEIRD Model benchmark without vaccines

Consider a homogeneous mixing of individuals within the population in a particular country, that is individuals in the population have an equal probability of contact with each other. For simplicity, we do not consider the effects of social distancing, lockdowns and other restrictive protocols implemented during COVID-19. Using a deterministic modelling approach to describe the disease transmission dynamics, the total population is subdivided into four different epidemiological compartments depending on individuals’ health status. The compartments of the SEIRD model are: S (Susceptible), E (Exposed), I (Infections), R (Recovered), and D (Dead). The flow chart of the SEIRD model is illustrated in Figure 4.

The system of partial differential equations for the SEIRD model depicted in Figure 4 is as follows:


(1) $$\begin{array}{l} \mathrm{dS}/\mathrm{dt}=-\beta \mathrm{SI}/N-\alpha S\\ \mathrm{dE}/\mathrm{dt}=\beta \mathrm{SI}/N-\eta E\\ \mathrm{dI}/\mathrm{dt}=\eta E-\gamma I\\ \mathrm{dR}/\mathrm{dt}=\gamma \left(1-\varphi \right)I\\ \mathrm{dD}/\mathrm{dt}=\gamma \varphi I \end{array}$$

Based on the differential system (1), the total population is thus given by N = S + E + I + R + D. The coefficient β represents the infection rate divided by N, that is a coefficient accounting for the susceptible people who get infected by infectious people, η represents the incubation rate for the transition from exposed to infected, φ is the mortality/death rate (the rate at which infectious individuals die from the disease) and γ represents the recovery rate (the rate at which infectious individuals recover).

### SEIRD Model with Vaccines

**Figure 5:**
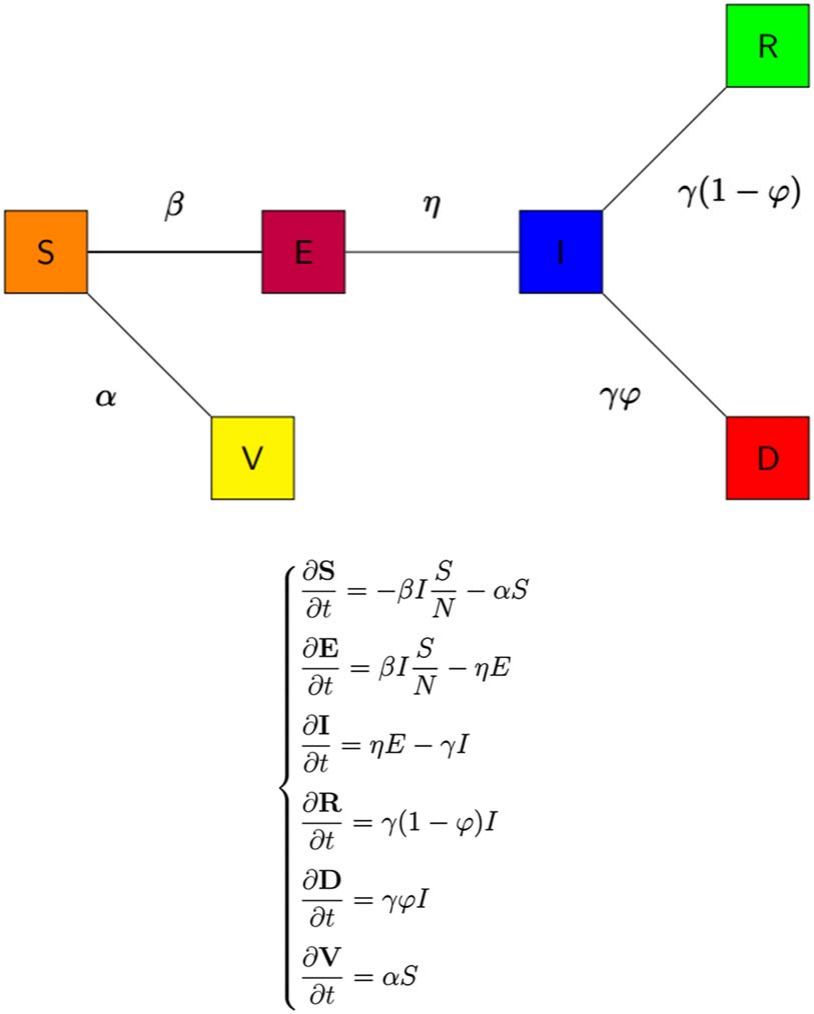
Illustration of the SEIRDV Model. Source: Authors illustration based on Piccolomini and Zama^26^.

It has been documented that countries are in a race to obtain COVID-19 vaccines for their populations^29^. There are two theoretical effects of vaccines documented in the literature, (i) a reduction in the pressure put on the healthcare infrastructure and personnel (this is due to the reduction of hospital quarantine and hospitalisations for severe cases) and a reduction in the rate of infections through the channel of herd immunity^14,30^. We examine an extended SEIRD model, which considers the vaccination of susceptible individuals hereafter the SEIRDV model (see Fig. 5). The compartments of the SEIRDV model are **S**usceptible, Exposed, Infections, Recovered, Dead, and Vaccinated.

From the idea presented by Piccolomini and Zama^11^, the following system of differential equations represents the dynamics of the population in the SEIRDV model:


(2) $$\begin{array}{l} \mathrm{dS}/\mathrm{dt}=-\beta \mathrm{SI}/N-\alpha S\\ \mathrm{dE}/\mathrm{dt}=\beta \mathrm{SI}/N-\eta E\\ \mathrm{dI}/\mathrm{dt}=\eta E-\gamma I\\ \mathrm{dR}/\mathrm{dt}=\gamma (1-\varphi )I\\ \mathrm{dD}/\mathrm{dt}=\gamma \varphi I\\ \mathrm{dV}/\mathrm{dt}=\alpha S \end{array}$$

The partial differential system of equations (2) is solved by starting from initial non-negative initial conditions S(0) = S0, E(0) = E0, I(0) = I0, Q(0) = Q0, R(0) = R0, D(0) = D0, and V(0) = V0. This means that the number of susceptible individuals, exposed individuals, exposed individuals, infectious individuals, recovered individuals, deceased individuals, and vaccinated individuals must be non-negative, respectively. The non-negative initial conditions ensure that the simulation of the SEIRDV model produces valid results. Negative initial conditions in any compartment are not physically valid and do not reflect the reality of the disease being modelled. For example, a negative starting condition for the infected individuals would imply that individuals get cured even before they get infected, which is impossible. In principle, initial conditions are the values of the compartments at the start of the simulation, before any infections and before the administration of vaccines. Total population size is given by N = S + E + I + R + D + V. As in the case of the benchmark model without the vaccine, the coefficients β, α, η, γ, φ, represent the infection rate divided by N, vaccination rate, incubation rate, recovery rate, and the death rate, respectively.

A critical parameter in epidemic modelling is the ‘basic reproduction rate (R_o_)’. It depends on the extent to which the virus is contagious and the number of contacts of an infected person. The size of the R_o_ will therefore vary from one country to another since its determination relies on averaging cases. It varies from country to country since various factors, such as population density, behaviour of individuals, and healthcare infrastructure influence it. Hypothetically, in a highly dense population, with less adherence to social distancing, the basic reproduction may be greater than in a sparsely populated country with a good functional healthcare system and adherence to health social distancing. Hethcote^31^ defined the reproduction rate as the expected number of secondary disease cases caused directly by a single infected individual introduced into a completely susceptible population.

It should be noted that there is currently no agreement in the literature regarding the length of time that COVID-19 vaccine immunity and effectiveness lasts. As a result, the study has made a simplifying assumption that all vaccinated individuals have indefinite immunity. Several African countries, such as South Africa and Tunisia, have not published statistical data on vaccine efficacy or immunity duration. Consequently, including an interaction between vaccination status and infection status in the model would be difficult to compute due to this limitation. The same simplifying assumption was made in a similar model by Antonelli *et al*.^32^. Hence, the model has no interaction between the vaccinated state and the infected state. Antonelli *et al*.^32^ presented an extension that illustrated the partial differential system, which captured the possibility of the vaccinated population being infected. The main challenge is identifying the duration of vaccine immunity, making the analysis very complex^33,34^. Likewise, there is no reliable source of data showing the proportion of infected individuals who were vaccinated.

## Data and data sources

The extended SEIRDV model was employed to forecast the COVID-19 pandemic in Tunisia and South Africa. When this study was carried out, Tunisia and South Africa had the most frequently reported data on vaccinations compared with many African countries. Moreover, we selected these two countries to provide variety in the size of the population, economic size as well as income classification according to the World Bank^30^. COVID-19 data was publicly available, freely accessible and downloaded from the Johns Hopkins Hospital website, this data included COVID-19 (confirmed, death, and recovered cases). The data were taken from 20-03-2021 to 30-12-2021.

COVID-19 vaccination data are obtained from Our World in Data website, which gives information about the total number of people who obtained atleast one dose of the vaccine as well as individuals who are fully vaccinated. Tunisia and South Africa considered in this study and selected based on the availability of data. The population data of the countries used in this study was obtained from the World Bank. The data do not show individuals who transition from being vaccinated to being infected, and neither does the data show the duration of immunity after vaccination.

Python 3.6 software was used to solve the system of ordinary differential equations, perform the simulations, and plot the fitted values that resulted from the model simulation and the actual data based on the COVID-19 case datasets. First, we simulate the model with some starting values of the main parameters.

## Results and analysis

We estimated the corresponding parameters of SEIRDV, by which the epidemic of COVID-19 can be modelled for Tunisia and South Africa with the respective standard errors displayed in Tables 1 and 2.

**Table 1:** Standard and relative errors for SEIRDV model for Tunisia

Parameter	Description	Value	Standard error	Relative error (%)	Initial value
Beta	Infection rate	5.32	0.00779	0.15	2.86
Alpha	Vaccination rate	0.00368	1.76	4.79	0.003
Eta	Incubation rate	1.49	0.0816	5.48	0.76
Varphi	Recovery rate	0.0530	0.00418	7.87	0.05

**Table 2:** Relative and standard errrors of the SEIRDV model for South Africa

Parameter	Description	Value	Standard error	Relative error (%)	Initial value
Beta	Infection rate	10.0	0.00413	0.04	9.65
Alpha	Vaccination rate	0.00847	3.91	4.61	0.0084
Eta	Incubation rate	1.06	0.104	9.78	0.86
Varphi	Recovery rate	0.0227	0.00206	9.07	0.02

**Figure 6:**
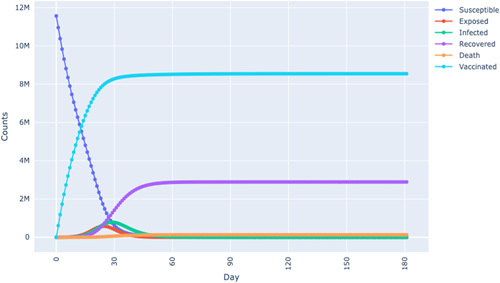
SEIRDV model simulation in Tunisia.

**Figure 7:**
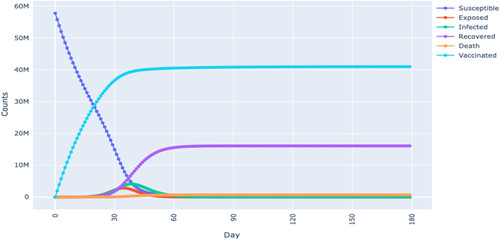
SEIRDV model simulation for South Africa.

In both Figures 6 and 7, a simulation was performed by increasing the vaccination rate, resulting in the flattening of the infection and mortality. This indicates the importance of vaccination in the evolution of transmission paths. Moreover, we also found that reducing the vaccination rate meant that the population depended more on obtaining herd immunity^14,30^. We discovered this point since a decline in the vaccination rate meant the recovery curve was rising higher. These findings are consistent with epidemiology literature in which either herd immunity or vaccination are used as tools to stem the transmission rates of a pandemic.

Table 1 illustrates the parameters of the model in column 1 and the respective description in column 2. The simulated values are shown in the third column with the standard error and relative errors of estimation of the parameters shown in column 4 and column 5, respectively. The initial parameter values used at the start of the simulation are indicated in the last column. All the figures in the table are written to three significant figures. The standard errors estimate the uncertainty associated with each parameter estimate. It is computed as the standard deviation of the sampling distribution of the estimated values of the parameters. Whereas the relative error is the expression of the standard error as a percentage of the estimated value. It gives an indication of the extent to which the parameter changed as a result of fitting the model to the data. The initial value is the starting point for the optimisation process or parameter fitting.

**Figure 8:**
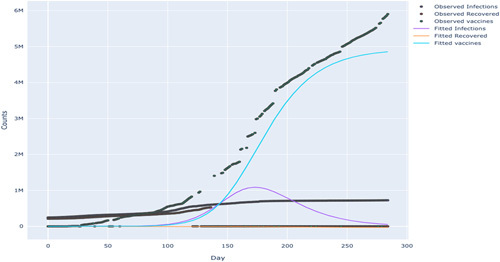
SEIRDV: observed vs. fitted model for Tunisia.

**Figure 9:**
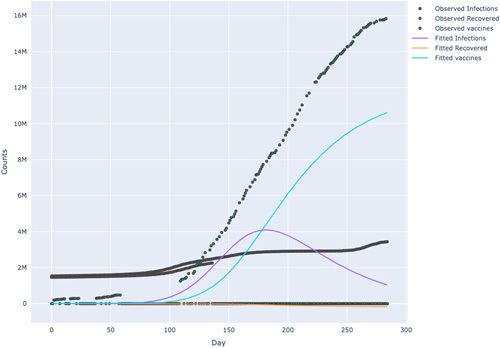
SEIRDV: observed vs. fitted model for South Africa.

As illustrated by Table 1, beta which represents the infection rate, is estimated to be 5.32, with a standard error of 0.00779 and a relative error of 0.15%. This means there is a relatively small amount of uncertainty in the estimated value. The starting point for parameter fitting used for beta is 2.86. It can be observed that the infection rate after vaccination shows an inverted U-shaped trend for both South Africa and Tunisia, as depicted in Figures 8 and 9. This implies that increasing the vaccination rates reduces the virus’s transmission rates and decreases mortality rates. This result is similar to Chen^14^, who studied the impact of vaccinations on COVID-19 infections in Israel, the UAE, Chile, the United Kingdom, Hungary, Qatar, Serbia, and the United States of America. The U-shaped trend characterises an increasing infection rate after vaccination up to a peak and then a decline with an increase in vaccine administration Chen^14^. Furthermore, the model fitted line compared with the actual data shows that the model approximates the data very well.

Table 2 summarises the starting values, relative errors of estimation and the final values of the model parameters of the SEIRDV model for South Africa. Analogous to Table 1, the interpretation of Table 2 provides an understanding of the evolution of the pandemic in South Africa. The infection rate (beta) is estimated at 10.0 (rounded to 3 significant figures) with a standard error of 0.00413 and a relative error of 0.04%. The low standard and relative errors indicate the high precision associated with the estimated infection rate.

## Discussion

The aim of this paper is to compare the model-predicted results with the available data to estimate the dynamics of the infected population, using data from 20-03-2021 to 30-12-2021. The analysis in Figures 8 and 9 showed that as the vaccine is rolled out, the cases of COVID-19 decline,that is the vaccine leads to a reduction in transmission or infection rate. This is a very important result. South Africa and Tunisia’s infection rate after vaccination shows an inverted U-shape. Furthermore, the model fitted line compared with the actual data shows that the model approximates the data very well.

In agreement with our finding, the WHO^34^ stated that “as Africa strives to step up vaccine uptake, it is witnessing a sustained decline in COVID-19 cases, the WHO further explained that Africa reported a 10% decline in COVID-19 cases for the week ending 13th March and that Deaths declined by 37% over the same period.”

However, this does not indicate that the pandemic has come to an end. Governments must continue to employ non-pharmaceutical containment protocols in order to ascertain that there is no resurgence of the pandemic. Moreover, the global fight against the pandemic can be a success when there is equitable distribution of vaccines, including scaling up vaccine uptake in African countries. For instance, it is crucial for developing nations to broaden their approach to vaccine distribution to ensure that their rural and isolated communities are not left behind. This can involve implementing large-scale vaccination drives and establishing robust community engagement initiatives that leverage the traditional structures unique to rural areas in Africa. With this approach, African countries can quickly roll-out the vaccine to a large proportion of their populations. Another important tenant in the success of vaccine roll-out in African countries is the political and leadership structures. A clearly planned and well-coordinated vaccination campaign requires significant funding for all operational costs.

## Conclusion

COVID-19 pandemic had devastating health and socioeconomic effects killing over 5 million people and reaching epidemic proportions worldwide. It is essential to model the spread and impact of the COVID-19 virus, which has led to significant efforts in epidemiology literature to forecast its trajectory and fit SIR and SEIR models. However, there has been a lack of attention in modelling vaccines, particularly in African countries. This research paper utilised a basic SEIRDV model to simulate infection and vaccination trajectories and tested its performance by comparing the fitted trajectory with actual data from the Johns Hopkins Hospital, which included counts of confirmed deaths, recovered cases, and vaccinated individuals.

The findings indicated that regardless of the specific type of vaccine utilised, administering the COVID-19 vaccine would be effective in curbing the pandemic’s spread and reducing infections. The study utilised data from Tunisia and South Africa, and the data showed a relatively good fit. These results are significant as they provide support for widespread vaccination efforts throughout Africa. They echo the sentiments expressed by the Regional Director for Africa of the World Health Organization, who emphasised that mass vaccination drives have generated a positive momentum in the fight against the pandemic on the continent. As more individuals receive vaccinations, the grip of COVID-19 on our lives weakens.

Although the model shows a relatively good fit with the data, there are multiple potential avenues for refining this system of differential equations to create a more accurate and intricate model. One possibility involves incorporating a transition from a vaccinated state to an exposed state, accounting for the subset of vaccinated individuals who may still contract and transmit the SARS-CoV-2 virus. Additionally, the model could integrate containment measures like lockdowns and social distancing. By implementing these sophisticated adjustments that more closely mirror reality, governments can utilise the model to better equip themselves for future outbreaks.

## Ethics approval

Not applicable.

## Consent for publication

Not applicable.

## Source of funding

We have not received any financial support for this manuscript.

## Author contribution

P.T., H.O., G.C.U. : conceptualization, project administration, writing—review and designing. All authors: data collection and assembly. P.T., H.O.: reviewed and edited the first draft. O.U.: reviewed and edited the second draft. P.T., H.O., G.C.U.: reviewed and edited the final draft. Manuscript writing: all authors. Final approval of manuscript: all authors.

## Conflicts of interest disclosure

The authors declare that there is no conflict/competition of interests.

## Research registration unique identifying number (UIN)

1. Name of the registry: Not Applicable.
2. Unique Identifying number or registration ID: Not Applicable.
3. Hyperlink to your specific registration (must be publicly accessible and will be checked): Not Applicable.

## Provenance and peer review

Not commissioned, externally peer-reviewed.

## Data availability statement

The datasets generated and/or analysed during the current study are available at the following weblinks: https://raw.githubusercontent.com/Lucas-Czarnecki/COVID-19-CLEANED-JHUCSSE/master/COVID-19_CLEAN/csse_covid_19_time_series_cleaned/time_series_covid19_cases_tidy.csv. This data source provides daily reported COVID-19 cases for a continuum of countries, including Tunisia and South Africa, used in this study. https://data.worldbank.org/. This data source provides the populations of a continuum of countries, including Tunisia and South Africa used in this study. Our World in Data: Coronavirus (COVID-19) Vaccinations - Our World in Data (accessed 24 April 24 2022). This data source provides the vaccination data for a continuum of countries, including Tunisia and South Africa, used in this study.

## Acknowledgements

The authors express their deep and sincere gratitude to the reviewers of this article.
